# Structures of Receptor Complexes of a North American H7N2 Influenza Hemagglutinin with a Loop Deletion in the Receptor Binding Site

**DOI:** 10.1371/journal.ppat.1001081

**Published:** 2010-09-02

**Authors:** Hua Yang, Li-Mei Chen, Paul J. Carney, Ruben O. Donis, James Stevens

**Affiliations:** Influenza Division, Centers for Disease Control and Prevention, Atlanta, Georgia, United States of America; Institut Pasteur, France

## Abstract

Human infections with subtype H7 avian influenza viruses have been reported as early as 1979. In 1996, a genetically stable 24-nucleotide deletion emerged in North American H7 influenza virus hemagglutinins, resulting in an eight amino acid deletion in the receptor-binding site. The continuous circulation of these viruses in live bird markets, as well as its documented ability to infect humans, raises the question of how these viruses achieve structural stability and functionality. Here we report a detailed molecular analysis of the receptor binding site of the North American lineage subtype H7N2 virus A/New York/107/2003 (NY107), including complexes with an avian receptor analog (3′-sialyl-N-acetyllactosamine, 3′SLN) and two human receptor analogs (6′-sialyl-N-acetyllactosamine, 6′SLN; sialyllacto-N-tetraose b, LSTb). Structural results suggest a novel mechanism by which residues Arg220 and Arg229 (H3 numbering) are used to compensate for the deletion of the 220-loop and form interactions with the receptor analogs. Glycan microarray results reveal that NY107 maintains an avian-type (α2-3) receptor binding profile, with only moderate binding to human-type (α2-6) receptor. Thus despite its dramatically altered receptor binding site, this HA maintains functionality and confirms a need for continued influenza virus surveillance of avian and other animal reservoirs to define their zoonotic potential.

## Author Summary

Influenza virus adaptation to different hosts usually results in a switch in receptor specificity of the viral surface coat protein, hemagglutinin. Indeed, the hemagglutinin subtypes from the last two human influenza pandemics of the 20^th^ Century (H2 in 1957 and H3 1968) both adapted successfully to human-type receptor specificity through only two amino acid mutations in the receptor binding pocket (Glutamine226→Leucine and Glycine228→Serine). The recent human infections reported with other avian subtypes such as H5, H7 and H9 have raised public health concerns and focused efforts on identifying potential subtypes from which a future pandemic strain may emerge. Since 1996, H7 viruses of the North American lineage have been circulating in regional live bird markets, containing an eight amino acid deletion in the receptor-binding site of HA. Here we report a detailed structural analysis of the receptor binding site of a hemagglutinin from the North American lineage of H7N2 viruses, in complex with avian and human receptor analogs, to understand how these viruses have adapted to such a dramatic structural change in the binding site while remaining one of the predominant circulating viral strains.

## Introduction

Influenza is an acute respiratory virus that infects up to 20% of the population in the United States, resulting in ∼36,000 deaths annually [1], [2]. The two membrane glycoproteins on the surface of influenza A virus, hemagglutinin (HA), which functions as the receptor binding and membrane fusion glycoprotein in cell entry, and neuraminidase (NA), which functions as the receptor destroying enzyme in virus release, form the basis for defining subtypes [3]. To date, 16 HA (H1–H16) and 9 NA (N1–N9) have been identified in avian species [4], while in the last century, only three subtypes, H1N1 in 1918 and 2009, H2N2 in 1957, and H3N2 in 1968 [5], [6], [7], have successfully adapted to humans. Hemagglutinin binds to sialic acid (SA) glycans present on host cell surfaces. The receptors on epithelial cells of the human upper respiratory tract are mainly α2-6-linked SA moieties [8]. Since avian influenza viruses predominately bind α2-3-linked SA, and human influenza viruses preferentially bind to α2-6-linked SA, human infection by avian influenza viruses is rare [9]. However, since 1997 a growing number of human cases of avian influenza infection have been reported [10], including H5N1, H7N2, H7N3, H7N7, and H9N2 strains [11]. Although the current situation with the pandemic H1N1 influenza virus dominates public health efforts, the prospect of a novel pandemic emerging from these isolated cases continues to be a major public health threat around the world.

Early cases of human infection by H7 influenza viruses are reported as far back as 1979 [12], [13]. Since 2002, multiple outbreaks and human infections of H7 subtype viruses; within both Eurasian and North American lineages have been reported. In the Netherlands in 2003, a highly pathogenic avian influenza (HPAI) H7N7 outbreak resulted in more than 80 cases of human infections, including one fatality [14], [15]. In New York in 2003, a single case of human respiratory infection of H7N2 was reported [16] and in British Columbia in 2004, an H7N3 virus caused two cases of conjunctivitis [17], [18]. More recently in 2007, the United Kingdom reported several cases of low pathogenic avian influenza (LPAI) H7N2 virus infections that caused influenza-like illness and conjunctivitis [19].

Since 1996, H7 viruses of the North American lineage have been circulating in regional live bird markets [20], containing a 24-nucleotide deletion resulting in an eight amino acid deletion in the receptor-binding site (RBS) of HA (Figure S1). The recent human infections with H7 in North America have raised public health concerns as to how these viruses adapt to such a dramatic structural change while remaining one of the predominant circulating viral strains. A recent study of H7 viruses isolated from previous outbreaks revealed efficient replication in both mouse and ferret animal models [21]. In particular, ferret studies with A/New York/107/2003 (NY107), an H7N2 virus isolated from a man in New York, not only showed efficient replication in the upper respiratory tract of the ferret but also the capacity for intra-species transmission by direct contact [21], [22]. Interestingly, both an increased preference for α2-6 and decreased preference for α2-3-linked sialosides of this virus compared to the other avian influenza viruses was shown by previous glycan microarray analysis but less so by a competitive solid-phase binding assay [22], [23].

Here we report a detailed molecular analysis of the RBS of the HA from North American lineage H7N2 virus, NY107, including glycan microarray analyses and structural analyses of the HA in complex with an avian receptor analog (3′-Sialyl-N-acetyllactosamine, 3′SLN) and two human receptor analogs (6′-Sialyl-N-acetyllactosamine, 6′SLN; Sialyllacto-N-tetraose b, LSTb). These results provide important insight into the interaction of H7 HAs with both avian and human hosts.

## Results

### Overall structure

**Figure 1:**
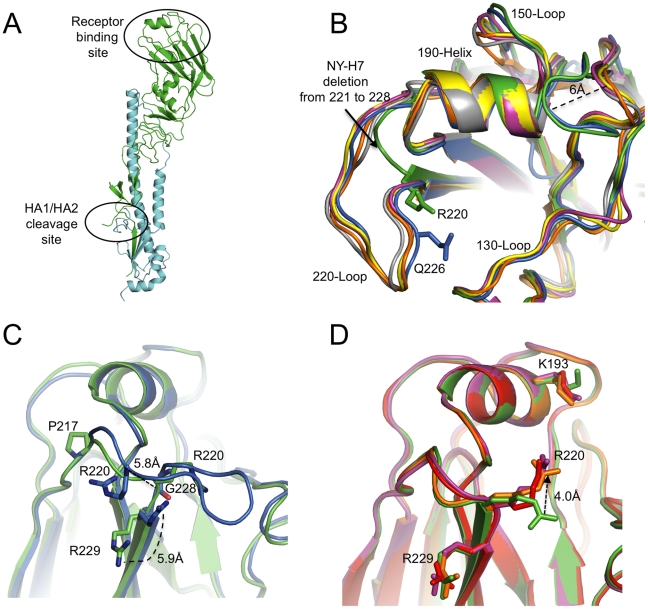
NY107 HA monomer and comparison of its RBS to other HA structures. (A) One monomer is shown with the HA1 chain colored in green and the HA2 chain in cyan. The location of the receptor binding site and the HA1/HA2 cleavage site are circled. (B) The superposition of receptor binding domains of NY107 (green), Av-H7 (marine), 1918-Hu-H1 (magenta), Hu-H5 (yellow), Hu-H3 (orange), and Sw-H9 (grey). The proximity of Arg220 and Gln226 are highlighted. Three structural elements comprising this binding site are labeled. The two major differences are the extended 150-loop and the deletion of 220-loop of NY107. (C) Overlap of NY107 (green) and Av-H7 (marine) (PDB: 1TI8) illustrates the compensatory effect of R220 bringing it close to the position occupied by G228 in the avian HA. (D) Overlap of the NY107 (green), NY107- 3′SLN (orange), NY107-6′SLN (red), and NY107-LSTb (magenta) structures. All the figures were generated and rendered with the use of MacPyMOL [56].

By using x-ray crystallography, the structure of H7 HA from the NY107 virus was determined to 2.6 Å resolution (Table 1). In addition, we also report three H7 HA receptor complex structures, with avian receptor analog (3′SLN) to 2.7 Å resolution and with human receptor analogs (6′SLN and LSTb) to 3.0 Å and 2.6 Å resolution, respectively (Table 1). The overall structure of NY107 is similar to other reported HA structures with a globular head containing the RBS and vestigial esterase domain, and a membrane proximal domain with its distinctive, central helical stalk and HA1/HA2 cleavage site (Figure 1A). Although five asparagine-linked glycosylation sites are predicted in the NY107 HA monomer, interpretable electron density was observed at only two sites, Asn38 in HA1 and Asn82 in HA2 (all residue numbers are based on H3 numbering). At these sites, only one or two N-acetyl glucosamines could be interpreted.

**Table 1:** Data collection and refinement statistics.

	NY107	NY107+3′SLN	NY107+6′SLN	NY107+LSTb
**Data collection**				
Space group	P2_1_2_1_2_1_	P2_1_2_1_2_1_	P2_1_2_1_2_1_	P2_1_2_1_2_1_
Cell dimensions (Å)	66.96, 115.92, 251.61	67.80, 116.70, 249.84	66.60, 116.58, 250.68	67.08, 116.52, 251.95
Resolution (Å)	50-2.6 (2.69-2.60)^a^	30-2.7 (2.80-2.70)	50-3.0 (3.11-3.0)	50-2.6 (2.69-2.60)
R_sym_ or R_merge_	10.6 (41.3)	14.6 (48.6)	14.3 (35.4)	12.2 (31.5)
I/σ	39.6 (2.0)	24.3 (1.7)	34.2 (8.2)	40.5 (9.9)
Completeness (%)	99.2 (99.0)	99.3 (94.6)	92.3 (75.6)	91.3 (86.2)
Redundancy	7.2 (6.2)	5.8 (5.5)	4.9 (4.4)	10.9 (11.2)
**Refinement**				
Resolution (Å)	50-2.6 (2.67-2.60)	30-2.7 (2.77-2.70)	50-3.0 (3.08-3.00)	50-2.6 (2.67-2.60)
No. of reflections (total)	57285	51770	33421	53603
No. of reflections (test)	3053	2769	1779	2842
R_work_/R_free_	21.7/25.6	21.4/26.4	20.5/26.0	20.4/24.7
No. of atoms	11795	11878	11648	12108
r.m.s.d.- bond length (Å)	0.006	0.006	0.008	0.006
r.m.s.d.- bond angle (°)	0.905	0.974	1.085	0.859
**MolProbity** b **scores**				
Favored (%)	96.9	96.5	94.3	97.1
Outliers (%) _(No. of residues)_	0.1 _(1/1434)_	0.0 _(0/1429)_	0.1 _(2/1433)_	0.1 _(2/1435)_

a Numbers in parentheses refer to the highest resolution shell.

b Reference [51].

During viral replication, HA is synthesized as a single chain precursor (HA0) and cleaved by specific host proteases into the infectious HA1/HA2 form. In baculovirus expression systems, highly pathogenic HAs, with a polybasic cleavage site, are expressed as an HA1/HA2 form [24], whereas HAs with monobasic cleavage sites (single Arg) from low pathogenic viruses are expressed as the HA0 form [25]. NY107 is regarded as a low pathogenic virus, and as expected, was produced in the HA0 form (Figure S2). However, subsequent digestion with thrombin protease to remove the His-tag resulted in cleavage to a profile on SDS-PAGE comparable to that of an HA1/HA2 form (Figure S2). A comparison of the NY107 cleavage site with the consensus cleavage pattern in the MEROPS database (http://merops.sanger.ac.uk) suggests it to be a possible thrombin cleavage site.

Based on their molecular phylogenies, HAs are divided into two groups and five clades: group 1 includes H8, H9, and H12; H1, H2, H5, and H6; H11, H13 and H16; group 2 includes H3, H4, and H14; H7, H10 and H15 [26]. Among all available HA structures, we selected ten representative HAs from both avian and human subtypes for structural analysis. As expected, NY107 HA is structurally very similar to the Avian-H7 in all comparisons and closely related to H3, the other group 2 members used in the analyses (Tables S1 and S2).

### The receptor binding site

The RBS is at the membrane distal end of each HA monomer and its specificity for sialic acid and the nature of its linkage to a vicinal galactose residue is a major determinant of host range-restriction. The consensus RBS for all current HAs is composed of three major structural elements: a 190-helix (residues 188–194), a 220-loop (residues 221–228), and a 130-loop (residues 134–138). In addition, highly conserved residues (Tyr98, Trp153, His183, and Tyr195) form the base of the pocket.

Although the NY107 RBS is similar to other subtypes (H1, H2, H3, H5, and H9), a previously observed specific feature of H7 HAs, is also observed in the NY107 150-loop region: two residues inserted at position 158 result in this loop protruding more than 6Å towards the binding site compared to other subtype HAs (Figure 1B and Table S2) [27]. More interestingly, the eight amino acid deletion, only found in the North American lineage H7s, from position 221 to 228 (Figure S1), resulted in a complete loss of the 220-loop (Figure 1B). Sequence alignment shows that Arg220 and Arg229 are conserved in all influenza A HA subtypes (Figure S1), but structural alignment of NY107 HA shows Arg220 occupying the Gly228 position, and the much shorter loop turns at residue Pro217 (Figure 1C). The Cα distance between NY107 Arg220 and its homolog in the Av-H7 structure (PDB: 1TI8) [27] is 5.8Å, and they point in opposite directions (Figure 1C). The side chain direction of Av-H7 Arg220 is almost parallel with the beta sheet after Arg229, whereas the NY107 Arg220 points downward to the binding pocket. The Cα position of Arg229 in both H7 structures remains the same, except the side chain in the NY107 swings away by about 5.9Å (Figure 1C) and could help to stabilize this region by forming a hydrogen bond to the mainchain carbonyl of Gln210 in the neighboring monomer. In the absence of the 220-loop in NY107 HA, upon glycan binding the long side chain of Arg220 compensates for its loss and is displaced 4Å upward to form hydrogen bonds with receptor analogs inside the binding pocket (Figure 1D).

### Effect of loop truncation on the receptor binding specificity of NY107

Previously, mutations in the HA receptor binding domains of H1N1 (Glu190Asp/Gly225Asp) and H2N2/H3N2 (Gln226Leu and Gly228Ser) subtypes were responsible for adaptation of these viruses to pandemic strains [24], [28], [29], [30]. Due to missing residues 221–228 in the NY107 HA RBS, neither mechanism for adaptation is possible. Thus, in order to look more closely at the role of the missing loop and its effect on receptor specificity, we first subjected the recombinant HA (recHA) to glycan microarray analyses and compared it to a reverse genetics-derived NY107 virus, and a co-circulating Eurasian virus and recHA, A/Netherlands/219/2003 (NL219), that has the consensus avian sequence in the 220-loop and it also infected a human [15].

**Figure 2:**
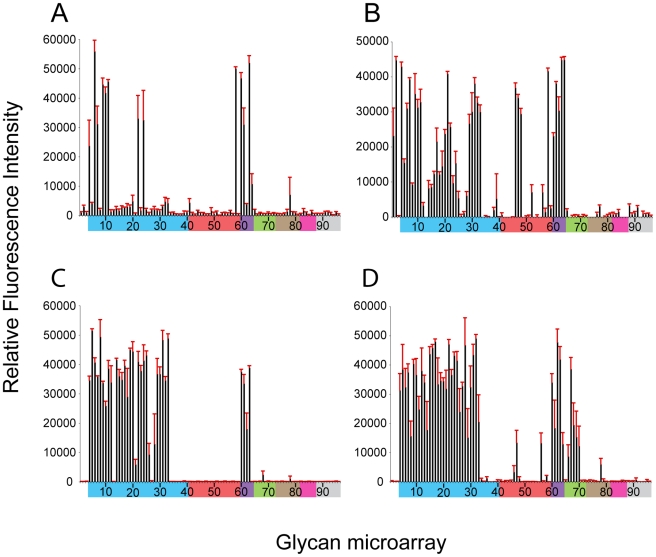
Receptor specificity of NY107 recHA and virus. Glycan microarray analysis of recombinant NY107 HA (A) and NY107 virus (B) compared to the recHA (C) and virus (D) from a Eurasian lineage A/Netherlands/219/2003 H7 influenza virus that was circulating in the same year and also infected a human. Colored bars highlight glycans that contain α2-3 SA (blue) and α2-6 SA (red), α2-6/α2-3 mixed SA (purple), N-glycolyl SA (green), α2-8 SA (brown), β2-6 and 9-O-acetyl SA, and non-SA (grey). Error bars reflect the standard error in the signal for six independent replicates on the array. Structures of each of the numbered glycans are found in Table S4.

Glycan microarray analysis of recombinant NY107 (Figure 2A and Table 2) revealed a highly restricted binding profile with strong binding to only α2-3 sulfated (#4–8), α2-3 branched (#9–11) and mixed α2-3/α2-6 branched sialosides (#60–64) as well as to the long linear sialyl di- and tri-lactosamines (#22, 24). Weak binding was also observed (above background) to other α2-3 glycans on the array. The recombinant NY107 also revealed a strict glycan binding preference to only one α2-6 glycan, the internal structure, Galβ1-3(Neu5Acα2-6)GlcNAcβ1-3Galβ1-4Glc (#58; LSTb) (Figure 2A), a glycan highlighted in a previous study [22]. The virus with higher valency and avidity revealed stronger binding to all α2-3 groups, in addition to the branched di-sialyl α2-6 biantennary structures (#46–48) as well the LSTb (#58) (Figure 2B and Table 2). In contrast, the NL219 recHA (Figure 2C and Table 2) bound well to only the avian α2-3 containing sialyl-glycans (sulfated, branched, linear and fucosylated). Its corresponding virus also reflected this specificity although it also revealed strong binding to α2-3 N-glycolylneuraminic acid (Neu5Gc) containing glycans (#66–70) (Figure 2D and Table 2).

**Table 2:** Comparison of the sialoside receptor specificity of the HAs from H7 influenza viruses.

Glycan Group	Graph Number^a^	NY107 RecHA	NY107 Virus	NY107-ins Virus	NY107-ins E186G Virus	NY107-ins R205G Virus	NY107-ins E186G/R205G Virus	NL219 RecHA	NL219 Virus
α**2-3**									
Sulfated	4–8	+++^b^	+++	+++	+++	+++	+++	+++	+++
Branched	9–11	+++	+++	+	+++	+++	+++	+++	+++
Linear	12–27	+	+++	+	+++	+++	+++	+++	+++
Fucosylated	28–34	−	+++	+++	+++	+++	+++	+++	+++
α**2-6**									
Sulfated	41	−	−	−	−	−	−	−	−
Branched mono-sialyl	42–45, 49	−	−	−	−	−	−	−	−
Branched di-sialyl	46–48	−	+++	−	−	−	−	−	−
Linear	50–56	−	−	−	−	−	−	−	−
Internal	58–59	+++	+++	−	−	−	−	−	−
**Other**									
Sialic acid	1–2	−	+++	+	−	−	−	−	−
α2-3/α2-6 Branched	60–64	−	−	−	+++	+++	+++	+++	+++
Neu5Gc^c^	65–72	−	−	−	+++	+++	+++	−	+++

a Members of each group are identified according to the graph number used in the microarray data in Figures 2 and 3 and correspond to numbers in the complete glycan list (Table S4).

b Binding of samples to glycan subclasses are qualitatively estimated based on relative strength of the signal for the data shown in Figures 2 and 3: strong (+++), weak (+), absent (−).

c N-glycolylneuraminic acid.

To further assess the effect of the missing 220 loop on HA structural stability and receptor specificity it was essential to evaluate these functions on the ancestral HA containing the full length 220-loop. To this end, we engineered an HA with an avian H7 consensus (PQVNGQSG) 220-loop re-introduced (NY107-220ins) into the NY107 HA and recovered this virus by reverse genetics. Compared to the NY107 virus (Figure 2A) glycan microarray analyses of the resulting NY107-220ins virus (Figure 3A and Table 2) revealed a decrease in binding to branched (#9–11) and linear (#12–27) α2-3 sialosides and a loss of binding to the branched di-sialyl α2-6 biantennary structures (#46–48), LSTb (#58) as well as the mixed α2-3/α2-6 branched sialosides (#60–64). In addition, sequence analysis of the NY107-220ins HA revealed the presence of quasispecies in the second position of the inserted loop, P(Q/K)VNGQSG, suggesting that re-introduction of the loop alone is not tolerated and does not create an avian-type binding profile. Thus other amino acid substitutions in the HA might have co-evolved with the deletion of the 220 loop to help stabilize the RBS/HA to maintain functionality.

**Figure 3:**
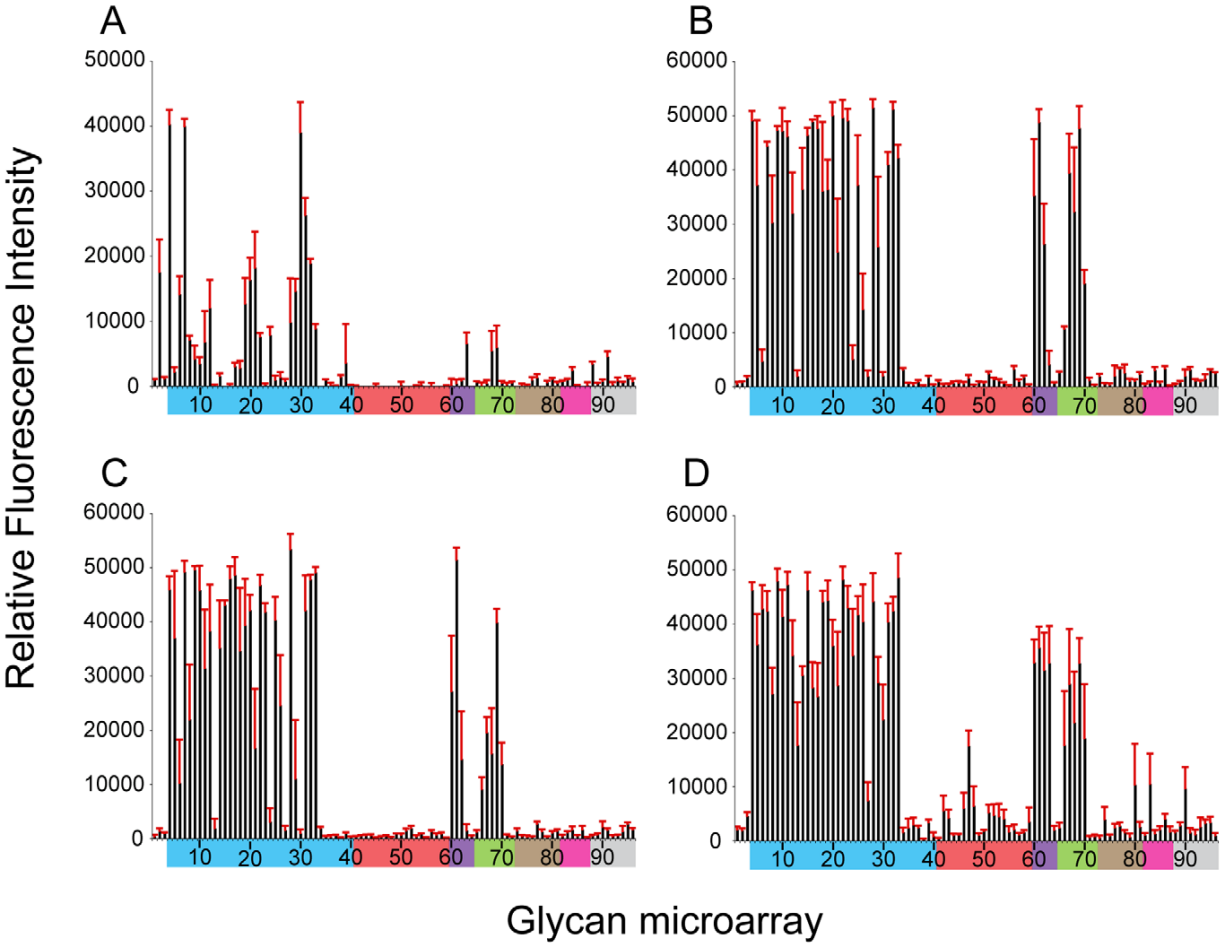
Effect of 220-loop deletion and additional RBS mutations on NY107 receptor specificity. NY107 was engineered to restore the 220-loop to a consensus full-length HA from 1996 (A) and additional co-variant amino acid substitutions, Glu186Gly (B), Arg205Gly (C) and the double mutant Glu186Gly/Arg205Gly (D) to restore, on the NY107 framework, an HA RBS found in viruses prior to the introduction of the deletion in North American viruses. Colored bars group glycans as described in Figure 3. Error bars reflect the standard error in the signal for six independent replicates on the array. Structures of each of the numbered glycans are found in Table S4.

When viruses containing this 220-loop deletion emerged in North America in the mid 90's, four additional amino acid substitutions, Gly114Arg, Asp119Gly, Gly186Glu and Gly205Arg, in the HA1 as well as an Asp19Asn in the HA2 chain were also introduced to most of the circulating isolates. Of these, Gly186Glu and Gly205Arg in the HA1 are close to the RBS, at the monomer interface, and could potentially modulate its structure and/or function. NY107 viruses with a restored consensus 220-loop and a single Glu186Gly (NY107-ins-186) or Arg205Gly (NY107-ins-205) substitution as well as the Glu186Gly/Arg205Gly double substitution (NY107-ins-186/205) were derived by reverse genetics and evaluated. Glycan microarray analysis for the three resulting viruses revealed similar glycan binding profiles with increased binding to α2-3 sialosides, including mixed α2-3/α2-6 branched sialosides (#60–64), α2-3 Neu5Gc (#66–70), but limited binding to the α2,6 sialosides (Figures 3B, 3C, 3D), resulting in a binding profile virtually identical to that of the NL219 virus and other avian influenza viruses (Figure 2D) [30]. Sequence analysis of the three reverse genetics derived viruses revealed no mutations/quasispecies in the HAs of either the NY107-ins-186 or the NY107-ins-186/205 virus stocks, indicative of replication fitness. For the NY107-ins-205 virus however, a Glu186Gly substitution emerged in the HA after only two passages in eggs following recovery from DNA transfection, indicating the importance of the co-variant position 186 with respect to HA functionality/glycan specificity. Altogether, the data indicates that the H7 subtype avian influenza viruses that were circulating in aquatic birds and poultry in North America before 1996 exhibited a classic avian α2-3 sialoside binding preference. In order for the 220-loop deletion to be tolerated, concurrent Gly186Glu and Gly205Arg substitutions in the vicinity of RBS of HA emerged to achieve a restricted α2-3 binding profile and only a moderate/limited increase in binding to branched di-sialyl α2-6 biantennary structures (#46–48) as well the α2,6 internal sialoside, LSTb (#58).

### NY107 avian receptor complex

**Figure 4:**
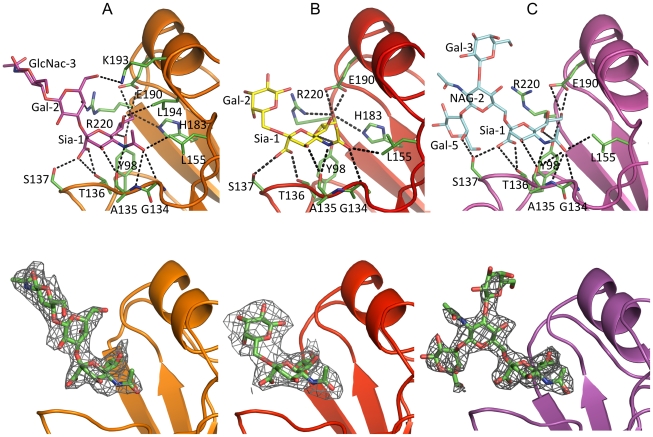
Glycan interactions within the NY107 RBS. The top panel shows the interactions of NY107 with (A) 3′SLN, (B) 6′SLN and (C) LSTb. NY107 is shown in orange/red/magenta cartoon respectively. The interacting HA residues are shown as green sticks. The bottom panel shows the electron density map of the ligands. The NY107 is shown in the same colors as above, and the ligands are shown as green sticks, the 2fo-fc electron density maps (contoured at 1σ) are shown in grey. Simulated annealing omit maps are shown in supplementary Figure S4.

To understand from a structural perspective how NY107 interacts with host receptors, we solved the structure of NY107 in complex with an avian and two human receptor analogs. For the avian receptor analog, 3′SLN, the electron density maps revealed well-ordered features for the Sia-1, Gal-2, and GlcNAc-3 in the NY107 HA complex structure (Figure 4A). Structural comparison of NY107 HA binding to other, H1, H2, H3, H5, and H9 subtypes (Figure S2A) revealed that 3′SLN binding to NY107 resembled binding of the other published HAs. Indeed, the terminal Sia-1 moiety is positioned almost identically in all structures, and forms the majority of hydrogen bonds and contacts with residues in the RBS (Figure 4A and Table S3).

Published avian HA structures with an intact 220-loop form very close interactions with Gal-2 of 3′SLN via residue Gln226 which is important in receptor specificity and host adaptation. For example, in the avian H7/3′SLN HA structure it interacts with Gal-2 O4 [31]. In the NY107 HA structure, although Gln226 is absent and no other residue occupies the same space as Gln226 (Figure 1B), Arg220 does forms a hydrogen bond between Arg220 NH2 and Gal-2 O4 (Figure 4A). Interestingly, although there was interpretable density for the GlcNAc-3 (Figure 4A and Figure S4B), no hydrogen bonding was apparent between the HA and the GlcNAc-3, which is consistent with other reported structures [32]. Thus, for binding to avian receptors, the *trans* conformation of α2-3 linkages is essential and perhaps only the first two saccharides are required. Indeed, due to the absence of 220-loop in the NY107 HA structure, the “aperture” of the RBS formed by 220-loop and 130-loop in regular HAs is increased by ∼10 Å, so that the branched, internal, and perhaps more complicated glycans might be accommodated more efficiently.

### NY107 human receptor complexes

In the NY107/6′SLN complex, only Sia-1 and Gal-2 are ordered (Figure 4B). The Sia-1 remains in the same position as previously analyzed glycan/HA complexes from H1, H2, H3, H5, and H9 (Figure S3B), whereas the Av-H7 complex structure with Sialyllacto-N-tetraose c (LSTc) did not reveal any density for the Sia-1 in the receptor binding site [31]. The Gal-2 position varies significantly among different subtypes. Compared to the human-adapted H1 HA [32], Gal-2 in the NY107 HA is 3Å higher, and thus is further from the protein (Figure S3B). In NY107, the Gal-2 only forms an intramolecular, saccharide-saccharide interaction with Sia-1. The poor electron density map and fewer interactions with protein residues suggest that the *cis* conformation of α2-6 linkages in 6′SLN trisaccharides show a reduced binding affinity with NY107.

Glycan array results with NY107 revealed a strong binding signal for the internal α2-6 sialoside, LSTb. To further investigate this interaction, we solved the structure of the NY107/LSTb complex. The final model contained Sia-1, NAG-2, Gal-3, and Gal-5 in the RBS. Although glycan microarray data indicated NY107 to have a specific affinity for LSTb, few interactions were apparent from the crystal structure. Sia-1 still forms multiple hydrogen bonds with residues in the RBS (Table S3 & Figure 4C). The branched Gal-5 interacts with Ser137, to help stabilize the LSTb binding. However, Arg220 and Lys193, the two residues showing close binding with 3′SLN, did not form any hydrogen bonds with LSTb. In the structure, Gal-5 also interacts with a crystal packing symmetry mate and thus the flexibility of whole LSTb may be restricted. In solution, with more freedom, the LSTb should be able to tilt closer to the RBS, and thus Glc-4 may have more interactions with the 190-helix than seen in the crystal structure.

## Discussion

Human infections by avian influenza viruses, including H7 subtypes, continue to pose a major public health threat. Although the species barrier prevents avian influenza viruses from widespread infection of the human population, the molecular determinants of efficient interspecies transmission and pathogenicity are still poorly understood. The viral coat protein HA however, is perhaps a critical molecule since previous pandemic viruses modified their receptor specificity and overcame the interspecies barrier to spread in the human population. Although HA structures alone and in complex with receptor analogs provide considerable insight into receptor binding, it is clear that HAs from different species and subtypes have significant structural variation. Indeed, low-pathogenic H7N2 avian influenza viruses with an 8 amino acid deletion within its RBS started to circulate in live-bird markets in the northeast United States in 1996. Despite what one would consider a debilitating mutation, these viruses have been reported as the predominant isolate [33]. Whether such a deletion contributed to their evolutionary success and how are an important questions, especially in light of NY107's ability to produce respiratory illness in humans [16], as well as its reported increased affinity for human-type receptors and ability for contact transmission in ferrets [21]. To try to help answer these questions, we have analyzed the molecular structures of NY107 and its complexes with receptor analogs to explain receptor specificity at the molecular level.

The crystal structures of NY107 and its complexes with both avian and human receptor analogs describe a mechanism as to how an influenza virus might adapt by dramatically altering its RBS, and still be functional. Arg220 of the HA1 chain of NY107 compensates for the loss of the 220-loop, by forming hydrogen bonds with Gal-2 from the avian analog (binding was not observed in either of the structures complexes with the human analogs). However, in the LSTb complex, branched Gal-5 forms extra interactions with the 130-loop, thus improving the binding preference for this particular glycan. Consistent with the structural evidence, glycan microarray analyses of NY107 revealed a strong binding preference for the branched α2-6 sialoside, LSTb. Except for the absence of the 220-loop, other key residues within the RBS are conserved in NY107 and thus, direct interactions with sialic acid are maintained.

The 220-loop is recognized as one of the three crucial structural elements in the RBS. Aside from the North American lineage H7N2 viruses, which have been circulating with a deletion (221–228) in this loop, there has been one other report describing a seven amino acid deletion (224–230) in a laboratory generated H3N2 escape mutant which was reported to have a slightly increased affinity for α2-3-linked glycans by hemagglutination assay [34]. Meanwhile, the equivalent region in the hemagglutinin-esterase-fusion (HEF) protein of influenza C virus reveals a rearrangement resulting in a truncated 260-loop in its RBS (Figure S5) [35]. However, without structural data with appropriate receptor analogs, it is not possible to compare the role of these loop variants in receptor binding to the H7 HA structure described here.

When compared to NL219, another co-circulating H7 avian virus HA (Figure 2C and D), overall binding to α2-3-linked glycans was markedly reduced, while increased binding to α2-6-linked receptors was only marginal. However, these results focus attention on only 2 sub-classes of human-type receptors that may be important for infection (and transmission in ferrets). The NY107 virus interaction with biantennary glycans (Figure 2B), although weak (not seen in Figure 2A with recHA), is a possible route for virus entry as biantennary structures are common on tissues, *i.e.* glycan profiling data from human lung tissue on the Consortium for Functional Glycomics (CFG) web site. In addition, the internal sialoside, LSTb, was observed in both virus and recHA microarray data, suggesting this type of glycan has good affinity for this HA. The significance of this is unknown since LSTb has only been described in human milk [36].

Interestingly, NY107 and NL219 virus receptor binding and specificity has been addressed previously using glycan microarray analysis that reported a significantly increased preference for α2-6 and decreased preference for α2-3-linked sialosides [22]. In addition, the same viruses were also included in a recent study from Gambaryan *et al.* using a competitive solid-phase binding assay [23]. Our findings confirm and extend the receptor binding specificity reported by these authors in that they reported both viruses binding to sulfated sialylglycans with a lactosamine (Galβ1-4GlcNAc core and reported only a moderate binding affinity for α2-6-sialyllactosamine, the human-type receptor analog used in their assay.

The 220-loop is an integral feature of the receptor binding site, and thus one would predict that such a deletion might have compromised this strain to be deleted from the population of circulating viruses. However, this was not the case [33] and its existence appears to be in part due to the additional mutations at positions 186 and 205. Restoration of the loop with either or both residues mutated back to the pre-1994 consensus sequence resulted in a classic avian influenza virus binding profile. The emergence of the Glu186Gly mutation in the HA of the NY107-ins-205 mutant after only two passages of the rescued virus in eggs, also indicates the importance of these positions for HA functionality/glycan specificity. Analysis of the structural data reveals that positions 186 and 205 are on opposite sides of a monomer but are both close to the 220-loop deletion region in the trimeric form. The Glu at position 186 is close to Arg220 and may interact with Arg220 when binding avian receptors. Position 205 in the neighboring monomer may be important in trimer stability and maintaining RBS functionality. If one models the pre-1996 220-loop restored into the NY107 structure, Arg205, Glu186 and the loop all clash, thus explaining the Glu186Gly mutation that emerged in the NY107-ins-205 virus HA after limited egg passage.

The NY107 RBS with its more restricted α2-3 glycan binding preference and weak/moderate increase in α2-6 binding may have given the virus a selective advantage to be maintained in poultry at live bird markets and supplying farms. Certain terrestrial birds, such as quails and chickens, have recently been shown to present both human and avian types of receptors in the trachea and intestine [37], [38], [39]. Although it is not known what specific glycans are presented in these animals, it is conceivable that a virus with mixed specificity might have a distinct advantage over avian viruses that have specific avian receptor requirements, particularly in bird markets where multiple species coalesce. Previous results with H7N2, H9N2 and H5N1 viruses all highlight the fact that an increase in α2-6-binding preference is not sufficient for efficient transmission of avian influenza viruses to humans [22], [40], [41]. Although it remains to be seen whether prolonged circulation of viruses in terrestrial birds, such as domestic chickens, can provide a possible route for viruses to adapt for efficient human infection [11], continued surveillance of influenza viruses from avian and other animal reservoirs is urgently needed to define their zoonotic potential.

## Materials and Methods

### Cloning

Based on H3 numbering [42], cDNA corresponding to residues 11–329 (HA1) and 1–176 (HA2) of the ectodomain of the hemagglutinin (HA) from A/New York/107/2003 (H7N2; Genbank:ACC55270) and A/Netherlands/219/2003 (H7N7; Genebank: AAR02640) was cloned into the baculovirus transfer vector, pAcGP67-A (BD Biosciences), incorporating a C-terminal thrombin cleavage site, a “foldon” sequence [43] and a His-tag at the extreme C-terminus of the construct to enable protein purification [25], [44]. Transfection and virus amplification were carried out according to the baculovirus expression system manual (BD Biosciences Pharmingen).

### Protein expression and purification

Soluble NY107 was recovered from the cell supernatant by metal affinity chromatography using Ni-NTA resin (Qiagen Inc.). Fractions containing NY107 were pooled and dialyzed against 10 mM Tris-HCl, 50 mM NaCl, pH 8.0, then subjected to ion-exchange chromatography (IEX) using a Mono-Q HR 10/10 column (GE Healthcare). IEX purified NY107 was subjected to thrombin digest (3 units/mg protein; overnight at 4°C) and purified by gel filtration chromatography using a Superdex-200 16/60 column (GE Healthcare) and 50 mM Tris-HCl, 100 mM NaCl, pH 8.0 as running buffer. Protein eluting as a trimer was buffer exchanged into 10 mM Tris-HCl, 50 mM NaCl, pH 8.0 and concentrated to 14.5 mg/ml for crystallization trials. At this stage, the protein sample still contained the additional plasmid-encoded residues at both the N (ADPG) and C terminus (SGRLVPR).

### Crystallization, ligand soaking and data collection

Initial crystallization trials were set up using a Topaz Free Interface Diffusion (FID) Crystallizer system (Fluidigm Corporation, San Francisco, CA). Crystals were observed in several conditions containing PEG 3350 or PEG 4000. Following optimization, diffraction quality crystals for NY107 were obtained at room temperature using a modified method for microbath under oil [45], by mixing the protein with reservoir solution containing 20% PEG 3350, 0.2 M magnesium chloride at pH 7.2. For receptor analog complexes, crystals were soaked for 3 hours in the crystallization buffer containing 10 mM 3′SLN or 6′SLN (V-labs Inc., Covington, LA), or overnight in 10mM LSTb (Sigma, St. Louis, MO). All crystals were flash-cooled at 100K using 20% glycerol as the cryo-protectant. Datasets were collected at Advanced Photon Source (APS) beamlines 22 ID and BM at 100K. Data were processed with the DENZO-SACLEPACK suite [46]. Statistics for data collection are presented in Table 1.

### Structure determination and refinement

The structure of NY107 was determined by molecular replacement with Phaser [47] using the structure of the avian H7 (Av-H7) from A/turkey/Italy/2002, pdb:1TI8 (HA1, 78% identity; HA2, 90% identity) as the searching model. One HA trimer occupies the asymmetric unit with an estimated solvent content of 58% based on a Matthews' coefficient (*Vm*) of 2.9 Å^3^/Da. Rigid body refinement of the trimer led to an overall R/Rfree of 28.6%/37.4%. The model was then “mutated” to the correct sequence and rebuilt by Coot [48], then the protein structures were refined with REFMAC [49] using TLS refinement [50]. The final models were assessed using MolProbity [51]. The three complex structures were refined and evaluated using the same strategy. All statistics for data processing and refinement are presented in Table 1. Electron density maps (2fo-fc) were generated in Refmac [49] while simulated annealing omit maps were generated by sa-omit-map, a part of the Crystallography and NMR System (CNS) software [52].

### Virus generation

Wild type and mutant viruses of NY107 (H7N2) and A/Netherland/219/2003 (H7N7) were generated from plasmids by a reverse genetics approach [53]. To generate viruses with amino acid insertion or substitution in the HA, mutations were introduced into plasmid DNA with an overlap extension PCR approach [54]. Viruses derived by plasmid transfection of HK293 cells were propagated in eggs. The genomes of resulting virus stocks were sequenced to detect the emergence of possible variants during amplification.

### Glycan binding analyses

Glycan microarray printing and recHA analyses have been described previously [24], [30], [44], [55] (see Table 2 for glycans used for analyses in these experiments). Virus were analyzed on the microarray as described previously [30].

### PDB accession codes

The atomic coordinates and structure factors of NY107 are available from the RCSB PDB under accession codes 3M5G for the unliganded NY107, 3M5H for the NY107 with 3′-SLN and 3M5I and 3M5J for NY107 with 6′SLN and LSTb, respectively.

### Accession/ID numbers for genes/proteins used in this work

A/New York/107/03 (H7N2), Genbank: ACC55270; A/Netherlands/219/03 (H7N7), Genbank: AAR02640; A/Hong Kong/1-9/68 (H3N2), 2HMG; A/Duck/Ukraine/1/63 (H3N8), PDB: 1MQL; A/South Carolina/1/18 (H1N1), PDB: 1RD8; A/Puerto Rico/8/34 (H1N1), PDB: 1RU7; A/Swine/Iowa/15/30 (H1N1), PDB: 1RUY; A/Singapore/1/1957 (H2N2), PDB: 2WRC; A/Viet Nam/1203/04 (H5N1), PDB: 2FK0; A/Duck/Singapore/3/97 (H5N3), PDB: 1JSM; A/Swine/Hong Kong/9/98 (H9N2), PDB: 1JSD; A/Turkey/Italy/8000/02 (H7N3), PDB: 1TI8; C/Johannesburg/1/66, 1FLC.

## Supporting Information

Figure S1 Sequence alignment of selected structurally available HAs. Human H3 (PDB: 2HMG), Avian H3 (PDB: 1MQL), 1918-Human H1 (PDB: 1RD8), 1934-Human H1 (PDB: 1RU7), Swine H1 (PDB: 1RUY), 1957-Huamn H2 (PDB: 2WRC), Human H5 (PDB: 2FK0), Avian H5 (PDB: 1JSM), Swine H9 (PDB: 1JSD), and Avian H7 (PDB: 1TI8) were used in the alignments. The fusion domain of HA1 is highlighted in magenta, the vestigial esterase domain is highlighted in green, the receptor binding domain is highlighted in blue, and the fusion domain of HA2 is highlighted in red. Residue numbering is based on the H3 HA sequence.
(2.84 MB TIF)

Figure S2 Expression and purification of NY107. SDS-PAGE reveals that NY107 was expressed as the HA0 form with a mass approximately 60kDa (middle lane). Thrombin cleavage resulted in an unexpected reduction in band size to a HA1/HA2 profile (right lane) with possible multiple glycoforms for the HA2 clearly present.
(0.23 MB TIF)

Figure S3 Comparison of glycan binding to NY107 with other HAs. A. Overlap of α2-3 ligands binding in the receptor binding site from NY-H7 (green), Av-H3 (orange), 1930-Hu-H1 (magenta), 1957-Hu-H2 (cyan), Av-H5 (yellow), and Sw-H9 (grey). B. Overlap of α2-6 linkage ligands binding in the receptor binding site from NY-H7 (green), Av-H3 (orange), 1930-Hu-H1 (magenta), 1957-Hu-H2 (cyan), Av-H5 (yellow), and Sw-H9 (grey).
(2.55 MB TIF)

Figure S4 Simulated annealing omit maps of the receptor binding site (contoured at 1σ). A. NY107 (blue), B. NY107-3′SLN (orange), C. NY107-6′SLN (red), and D. NY107-LSTb (magenta). The protein model is shown in cartoon, and the residues involved in the binding to receptor analogs were shown in sticks. Maps were generated using version 1.2 of the Crystallography and NMR System (CNS) software.
(1.93 MB TIF)

Figure S5 Comparison of NY107 RBS to HEF. Overlap of RBS from NY107 (green), Av-H7 (marine) and HEF (magenta).
(1.12 MB TIF)

Table S1 Comparison of r.m.s.d. (Å) for different HA domains. For analyzing differences in the overall structure, r.m.s.d. values were calculated between monomers or domains of different HA's, after the Cα atoms of the HA2 domains were superposed by sequence and structural alignment onto the equivalent domains of NY107.
(0.04 MB DOC)

Table S2 Comparison of r.m.s.d. (Å) for individual domains. Each domain was superimposed separately to determine how the individual NY107 domains compared to equivalent domains in the other structures.
(0.04 MB DOC)

Table S3 Molecular interactions between NY107 and receptor analogs. The hydrogen bond cutoff is 3.8 Å for the listing interactions.
(0.07 MB DOC)

Table S4 Glycan array differences between NY107, the fully restored NY107-ins, and NL219 (virus and rHA). The color coding in the left hand column reflects the same coloring scheme used in Figures 2 and 3. Significant binding of samples to glycans are qualitatively estimated based on relative strength of the signal for the data shown in Figures 2 and 3 Strong (+++), weak (+).
(0.19 MB DOC)
