# Supplementary figures and images for: Structures of Receptor Complexes of a North American H7N2 Influenza Hemagglutinin with a Loop Deletion in the Receptor Binding Site
Source: PLoS Pathog. 2010 Sep 2;6(9):e1001081. doi: 10.1371/journal.ppat.1001081 (PMC2932715; doi:10.1371/journal.ppat.1001081)

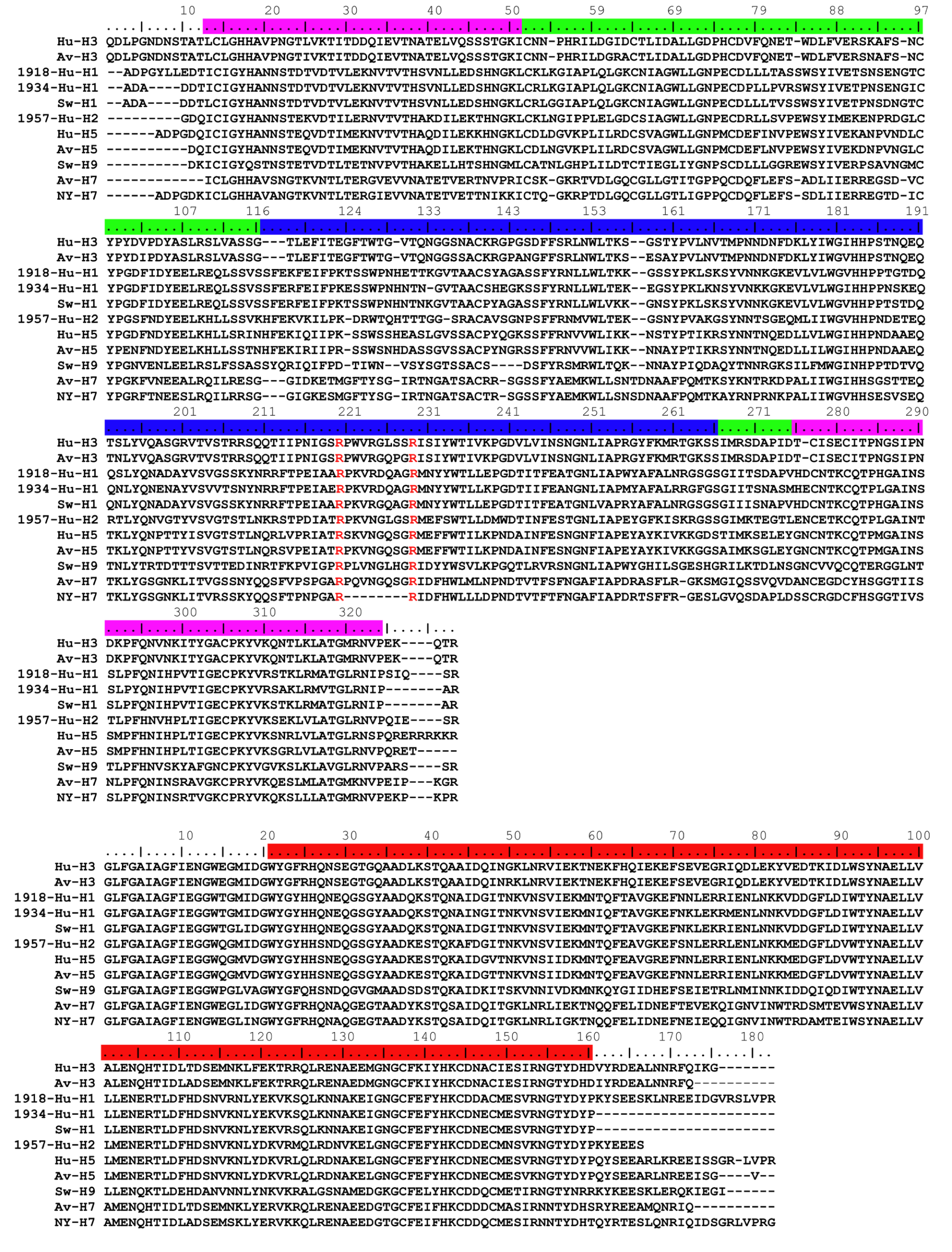

Supplement: Figure S1 — Sequence alignment of selected structurally available HAs. Human H3 (PDB: 2HMG), Avian H3 (PDB: 1MQL), 1918-Human H1 (PDB: 1RD8), 1934-Human H1 (PDB: 1RU7), Swine H1 (PDB: 1RUY), 1957-Huamn H2 (PDB: 2WRC), Human H5 (PDB: 2FK0), Avian H5 (PDB: 1JSM), Swine H9 (PDB: 1JSD), and Avian H7 (PDB: 1TI8) were used in the alignments. The fusion domain of HA1 is highlighted in magenta, the vestigial esterase domain is highlighted in green, the receptor binding domain is highlighted in blue, and the fusion domain of HA2 is highlighted in red. Residue numbering is based on the H3 HA sequence. (2.84 MB TIF) [file ppat.1001081.s001.tif]

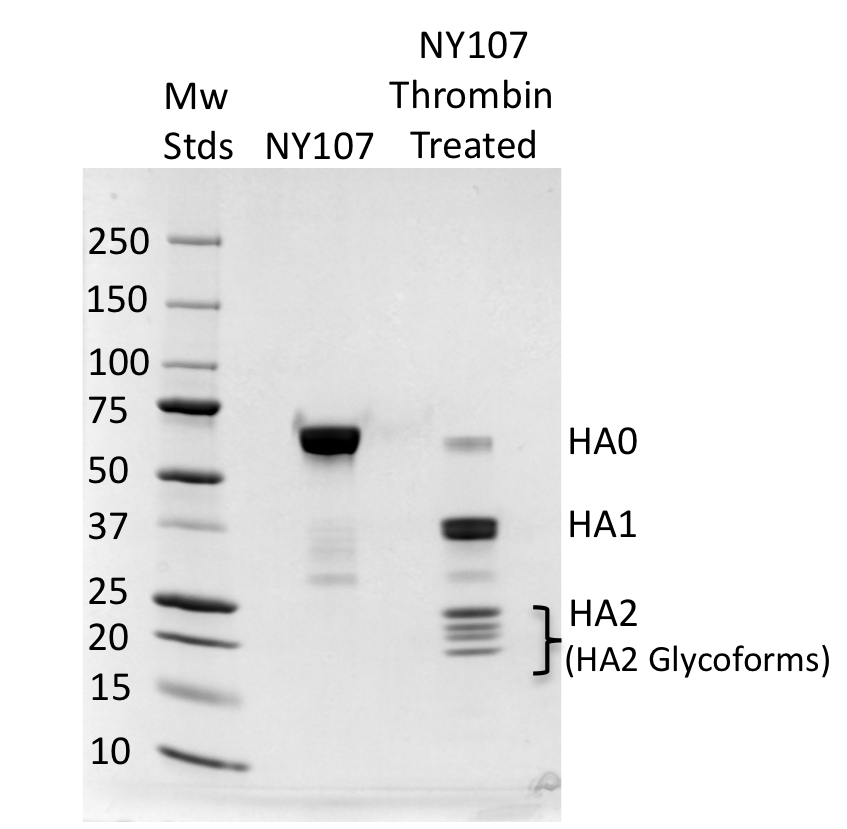

Supplement: Figure S2 — Expression and purification of NY107. SDS-PAGE reveals that NY107 was expressed as the HA0 form with a mass approximately 60kDa (middle lane). Thrombin cleavage resulted in an unexpected reduction in band size to a HA1/HA2 profile (right lane) with possible multiple glycoforms for the HA2 clearly present. (0.23 MB TIF) [file ppat.1001081.s002.tif]

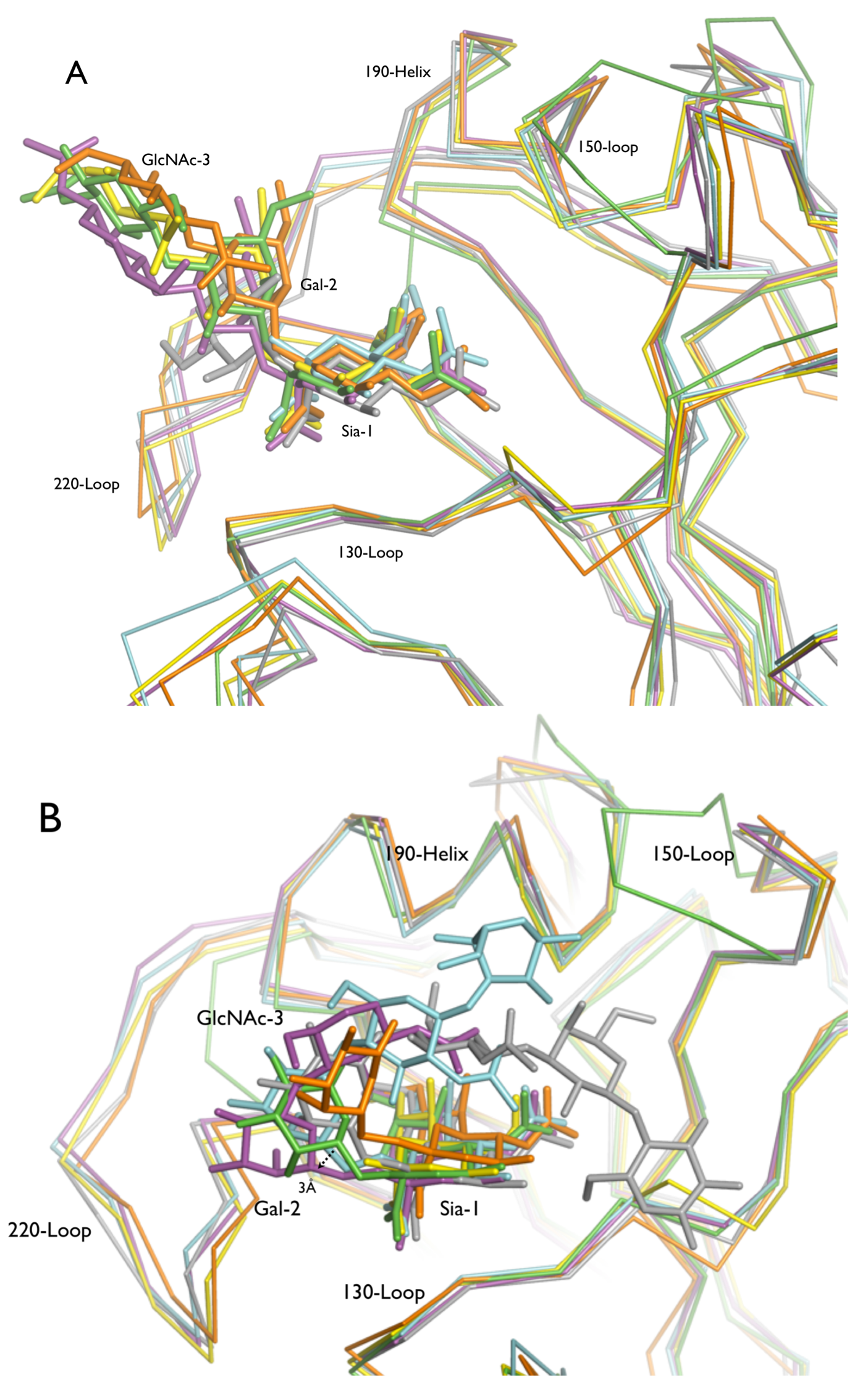

Supplement: Figure S3 — Comparison of glycan binding to NY107 with other HAs. A. Overlap of α2-3 ligands binding in the receptor binding site from NY-H7 (green), Av-H3 (orange), 1930-Hu-H1 (magenta), 1957-Hu-H2 (cyan), Av-H5 (yellow), and Sw-H9 (grey). B. Overlap of α2-6 linkage ligands binding in the receptor binding site from NY-H7 (green), Av-H3 (orange), 1930-Hu-H1 (magenta), 1957-Hu-H2 (cyan), Av-H5 (yellow), and Sw-H9 (grey). (2.55 MB TIF) [file ppat.1001081.s003.tif]

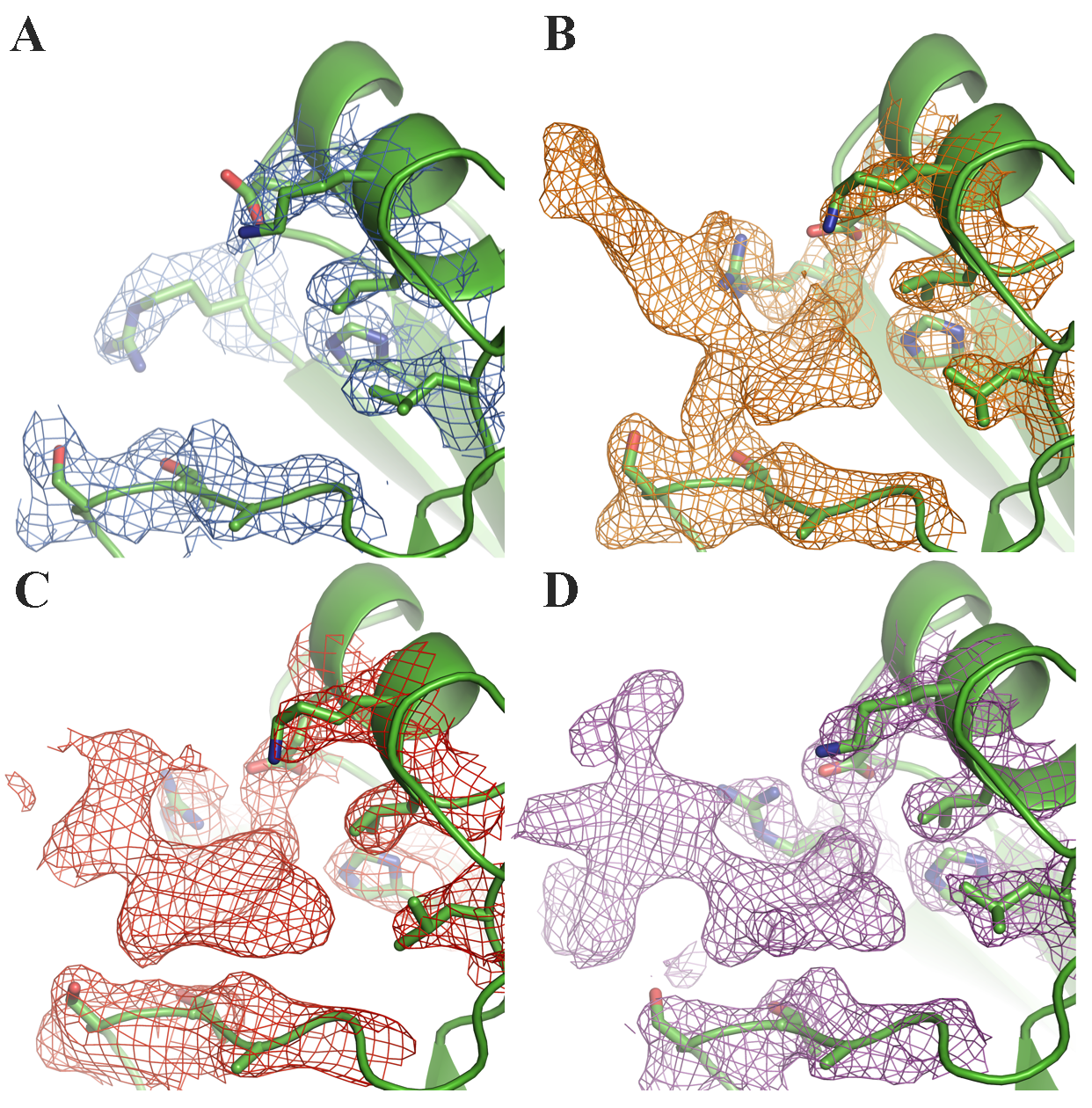

Supplement: Figure S4 — Simulated annealing omit maps of the receptor binding site (contoured at 1σ). A. NY107 (blue), B. NY107-3′SLN (orange), C. NY107-6′SLN (red), and D. NY107-LSTb (magenta). The protein model is shown in cartoon, and the residues involved in the binding to receptor analogs were shown in sticks. Maps were generated using version 1.2 of the Crystallography and NMR System (CNS) software. (1.93 MB TIF) [file ppat.1001081.s004.tif]

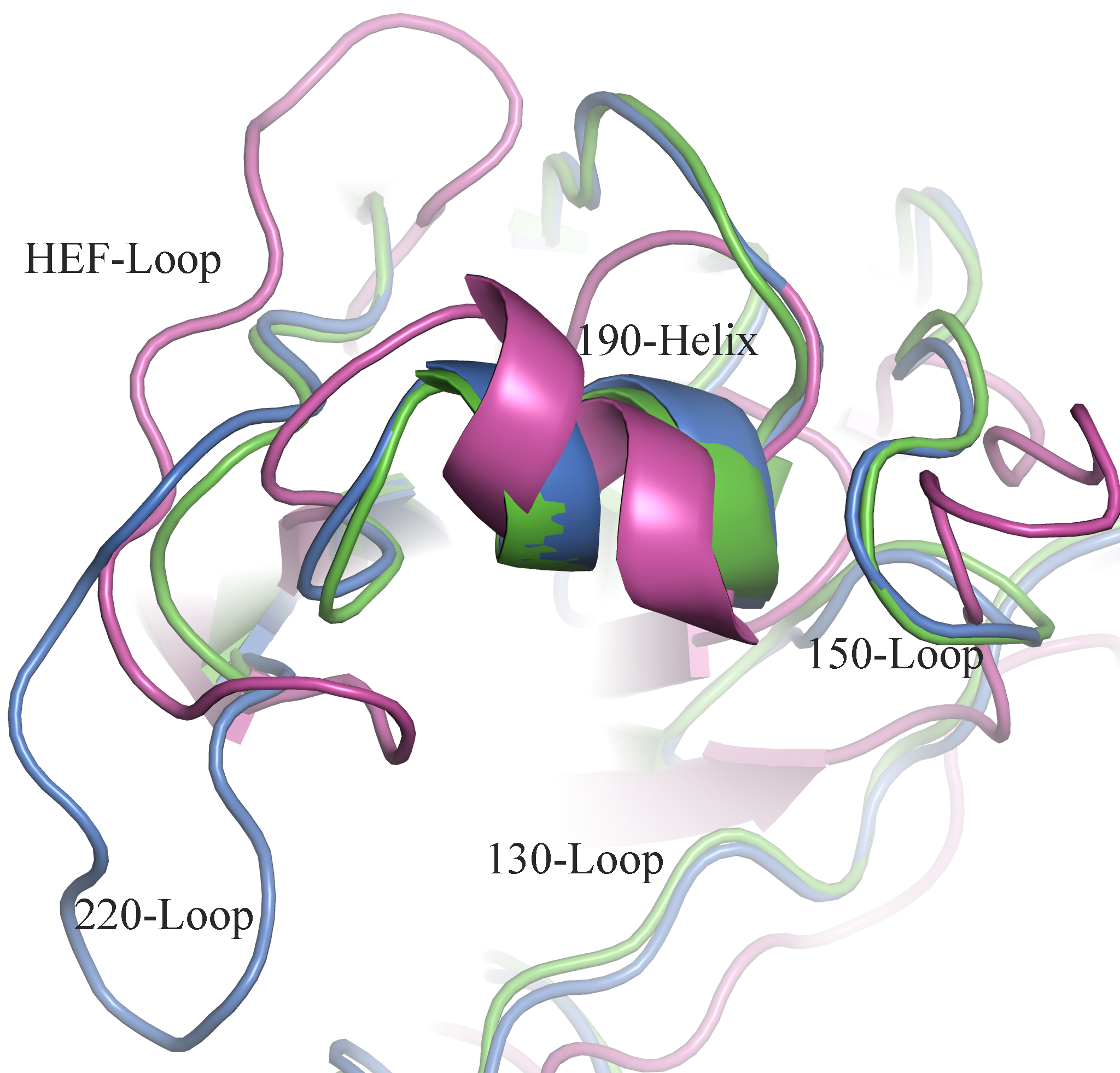

Supplement: Figure S5 — Comparison of NY107 RBS to HEF. Overlap of RBS from NY107 (green), Av-H7 (marine) and HEF (magenta). (1.12 MB TIF) [file ppat.1001081.s005.tif]
