# Supplementary material for: Structures of Receptor Complexes of a North American H7N2 Influenza Hemagglutinin with a Loop Deletion in the Receptor Binding Site
Source: PLoS Pathog. 2010 Sep 2;6(9):e1001081. doi: 10.1371/journal.ppat.1001081 (PMC2932715; doi:10.1371/journal.ppat.1001081)
Supplement: Table S1 — Comparison of r.m.s.d. (Å) for different HA domains. For analyzing differences in the overall structure, r.m.s.d. values were calculated between monomers or domains of different HA's, after the Cα atoms of the HA2 domains were superposed by sequence and structural alignment onto the equivalent domains of NY107. (0.04 MB DOC) [file ppat.1001081.s006.doc]

**Table S1.** **Comparison of r.m.s.d. (Å) for different HA domains.** For analyzing differences in the overall structure, r.m.s.d. values were calculated between monomers or domains of different HA’s, after the Cα atoms of the HA2 domains were superposed by sequence and structural alignment onto the equivalent domains of NY107.

| Group | Subtype | PDB entry | Monomer | HA1 Domain | HA1 “R” Region | HA1 “E” Region |
| --- | --- | --- | --- | --- | --- | --- |
| 1 | Human H1N1  South Carolina/1/1918 | 1RD8 | 5.19 | 7.13 | 7.62 | 6.12 |
| 1 | 1934-Hu-H1N1  Puerto Rico/8/34 | 1RU7 | 5.10 | 6.85 | 7.28 | 5.97 |
| 1 | Swine H1N1  swine/Iowa/30, | 1RUY | 4.65 | 6.13 | 6.33 | 5.74 |
| 1 | 1957-Hu-H2N2  Singapore/ 1/57 | 2WRC | 7.68 | 9.04 | 9.92 | 7.15 |
| 1 | Hu-H5N1  Vietnam/1203/2004 | 2FK0 | 5.41 | 7.48 | 8.21 | 5.92 |
| 1 | Av-H5N1  duck/Singapore/3/1997 | 1JSM | 4.71 | 6.15 | 6.40 | 5.67 |
| 1 | Sw-H9N2  swine/Hong Kong/9/1998 | 1JSD | 3.98 | 5.14 | 5.24 | 4.95 |
| 2 | Human H3N2  HongKong/19/1968 | 2HMG | 2.22 | 2.74 | 2.87 | 2.49 |
| 2 | Avian H3N2  duck/Ukraine/1963 | 1MQL | 2.40 | 3.05 | 3.26 | 2.64 |
| 2 | Av-H7N3  turkey/Italy/2002 | 1TI8 | 1.69 | 2.31 | 2.76 | 1.02 |
