# Supplementary material for: Structures of Receptor Complexes of a North American H7N2 Influenza Hemagglutinin with a Loop Deletion in the Receptor Binding Site
Source: PLoS Pathog. 2010 Sep 2;6(9):e1001081. doi: 10.1371/journal.ppat.1001081 (PMC2932715; doi:10.1371/journal.ppat.1001081)
Supplement: Table S2 — Comparison of r.m.s.d. (Å) for individual domains. Each domain was superimposed separately to determine how the individual NY107 domains compared to equivalent domains in the other structures. (0.04 MB DOC) [file ppat.1001081.s007.doc]

**Table S2.** **Comparison of r.m.s.d. (Å) for individual domains.** Each domain was superimposed separately to determine how the individual NY107 domains compared to equivalent domains in the other structures.

| Group | Subtype | PDB entry | HA2 Domain | HA1 Domain | HA1 “R” Region | HA1 “E” Region |
| --- | --- | --- | --- | --- | --- | --- |
| 1 | Human H1N1  South Carolina/1/1918 | 1RD8 | 2.15 | 2.09 | 1.64 | 2.53 |
| 1 | 1934-Hu-H1N1  Puerto Rico/8/34 | 1RU7 | 2.53 | 1.61 | 1.39 | 1.77 |
| 1 | Swine H1N1  swine/Iowa/30, | 1RUY | 2.62 | 1.65 | 1.40 | 1.83 |
| 1 | 1957-Hu-H2N2  Singapore/ 1/57 | 2WRC | 2.77 | 3.80 | 3.54 | 2.56 |
| 1 | Hu-H5N1  Vietnam/1203/2004 | 2FK0 | 2.07 | 2.01 | 1.62 | 2.44 |
| 1 | Av-H5N1  duck/Singapore/3/1997 | 1JSM | 2.75 | 1.52 | 1.26 | 1.82 |
| 1 | Sw-H9N2  swine/Hong Kong/9/1998 | 1JSD | 2.46 | 1.54 | 1.39 | 1.66 |
| 2 | Human H3N2  HongKong/19/1968 | 2HMG | 1.58 | 1.66 | 1.46 | 1.48 |
| 2 | Avian H3N2  duck/Ukraine/1963 | 1MQL | 1.54 | 1.65 | 1.46 | 1.50 |
| 2 | Av-H7N3  turkey/Italy/2002 | 1TI8 | 0.71 | 0.70 | 0.70 | 0.48 |
