# Supplementary material for: Structures of Receptor Complexes of a North American H7N2 Influenza Hemagglutinin with a Loop Deletion in the Receptor Binding Site
Source: PLoS Pathog. 2010 Sep 2;6(9):e1001081. doi: 10.1371/journal.ppat.1001081 (PMC2932715; doi:10.1371/journal.ppat.1001081)
Supplement: Table S3 — Molecular interactions between NY107 and receptor analogs. The hydrogen bond cutoff is 3.8 Å for the listing interactions. (0.07 MB DOC) [file ppat.1001081.s008.doc]

**Table S3.** **Molecular interactions between NY107 and receptor analogs.** The hydrogen bond cutoff is 3.8 Å for the listing interactions.

| **3’SLN** | | **Protein** | |
| --- | --- | --- | --- |
| SIA1 | O10 | L155 | CD2 |
|  | O10 | A135 | m/c N |
|  | O10 | G134 | m/c Ca |
|  | O9 | H183 | NE2 |
|  | O9 | E190 | OE1 |
|  | O9 | E190 | OE2 |
|  | O9 | Y98 | OH |
|  | O8 | Y98 | OH |
|  | O7 | L194 | CD2 |
|  | O1A | S137 | OG |
|  | O1A | T136 | OG1 |
|  | O1B | T136 | OG1 |
|  | N5 | A135 | m/c O |
|  | O4 | A135 | m/c O |
| Gal2 | O4 | R220 | NH2 |
|  | O6 | K193 | NZ |

| **6’SLN** | | **Protein** | |
| --- | --- | --- | --- |
| SIA1 | O10 | L155 | CD2 |
|  | O10 | G134 | m/c Ca |
|  | O9 | H183 | NE2 |
|  | O9 | E190 | OE1 |
|  | O9 | E190 | OE2 |
|  | O9 | R220 | NH1 |
|  | O8 | R220 | NH1 |
|  | O9 | Y98 | OH |
|  | O8 | Y98 | OH |
|  | O1A | S137 | OG |
|  | O1B | T136 | OG1 |
|  | N5 | A135 | m/c O |

| **LSTb** | | **Protein** | |
| --- | --- | --- | --- |
| SIA1 | O10 | L155 | CD2 |
|  | O10 | A135 | m/c N |
|  | O10 | A135 | m/c O |
|  | O10 | G134 | m/c Ca |
|  | O9 | H183 | NE2 |
|  | O9 | E190 | OE1 |
|  | O9 | E190 | OE2 |
|  | O9 | Y98 | OH |
|  | O8 | Y98 | OH |
|  | O1A | S137 | OG |
|  | O1A | T136 | OG1 |
|  | O1B | T136 | OG1 |
|  | N5 | A135 | m/c O |
|  | O4 | A135 | m/c O |
| Gal5 | O4 | S137 | OG |
