# Supplementary material for: Structures of Receptor Complexes of a North American H7N2 Influenza Hemagglutinin with a Loop Deletion in the Receptor Binding Site
Source: PLoS Pathog. 2010 Sep 2;6(9):e1001081. doi: 10.1371/journal.ppat.1001081 (PMC2932715; doi:10.1371/journal.ppat.1001081)
Supplement: Table S4 — Glycan array differences between NY107, the fully restored NY107-ins, and NL219 (virus and rHA). The color coding in the left hand column reflects the same coloring scheme used in Figures 2 and 3. Significant binding of samples to glycans are qualitatively estimated based on relative strength of the signal for the data shown in Figures 2 and 3 Strong (+++), weak (+). (0.19 MB DOC) [file ppat.1001081.s009.doc]

**Table S4.** **Glycan array differences between NY107, the fully restored NY107-ins, and NL219 (virus and rHA).** The color coding in the left hand column reflects the same coloring scheme used in Figures 2 and 3. Significant binding of samples to glycans are qualitatively estimated based on relative strength of the signal for the data shown in Figures 2 and 3 Strong (+++), weak (+).

| **#** | **Structure** | **NY107 RecHA** | **NY107 Virus** | **NY107-ins Virus** | **NY107-ins E186G Virus** | **NY107-ins R205G Virus** | **NY107-ins E186G/ R205G Virus** | **NL219 RecHA** | **NL219 Virus** |
| --- | --- | --- | --- | --- | --- | --- | --- | --- | --- |
| 1 | α-Neu5Ac |  | +++ |  |  |  |  |  |  |
| 2 | α-Neu5Ac |  | +++ | +++ |  |  |  |  |  |
| 3 | β-Neu5Ac-Sp8 |  |  |  |  |  |  |  |  |
| 4 | Neu5Acα2-3(6-O-Su)Galβ1-4(Fucα1-3)GlcNAc | +++ | +++ | +++ | +++ | +++ | +++ | +++ | +++ |
| 5 | Neu5Acα2-3Galβ1-3[6OSO3]GalNAc |  | +++ |  | +++ | +++ | +++ | +++ | +++ |
| 6 | Neu5Acα2-3Galβ1-4[6OSO3]GlcNAc | +++ | +++ | +++ |  |  | +++ | +++ | +++ |
| 7 | Neu5Acα2-3Galβ1-4(Fucα1-3)(6OSO3)GlcNAc | +++ | +++ | +++ | +++ | +++ | +++ | +++ | +++ |
| 8 | Neu5Acα2-3Galβ1-3(6OSO3)GlcNAc |  | + | + | +++ | +++ | +++ | +++ | +++ |
| 9 | Neu5Acα2-3Galβ1-3(Neu5Acα2-3Galβ1-4)GlcNAc | +++ | +++ | + | +++ | +++ | +++ | +++ | +++ |
| 10 | Neu5Acα2-3Galβ1-3(Neu5Acα2-3Galβ1-4GlcNAcβ1-6)GalNAc | +++ | +++ | + | +++ | +++ | +++ | +++ | +++ |
| 11 | Neu5Acα2-3Galβ1-4GlcNAcβ1-2Manα1-3(Neu5Acα2-3Galβ1-4GlcNAcβ1­2Manα1-6)Manβ1-4GlcNAcβ1-4GlcNAc | +++ | +++ | + | +++ | +++ | +++ | +++ | +++ |
| 12 | Neu5Acα2-3Gal |  |  | + | +++ | +++ | +++ | +++ | +++ |
| 13 | Neu5Acα2-3GalNAc |  |  |  |  |  | +++ |  | +++ |
| 14 | Neu5Acα2-3Galβ1-3GalNAc |  | + |  | +++ | +++ | +++ | +++ | +++ |
| 15 | Neu5Acα2-3Galβ1-3GlcNAc |  | + |  | +++ | +++ | +++ | +++ | +++ |
| 16 | Neu5Acα2-3Galβ1-3GlcNAc |  | + |  | +++ | +++ | +++ | +++ | +++ |
| 17 | Neu5Acα2-3Galβ1-4Glc |  | +++ |  | +++ | +++ | +++ | +++ | +++ |
| 18 | Neu5Acα2-3Galβ1-4Glc |  | + |  | +++ | +++ | +++ | +++ | +++ |
| 19 | Neu5Acα2-3Galβ1-4GlcNAc |  | + | +++ | +++ | +++ | +++ | +++ | +++ |
| 20 | Neu5Acα2-3Galβ1-4GlcNAc |  | +++ | +++ | +++ | +++ | +++ | +++ | +++ |
| 21 | Neu5Acα2-3GalNAcβ1-4GlcNAc |  | +++ | +++ | +++ | +++ | +++ | + | +++ |
| 22 | Neu5Acα2-3Galβ1-4GlcNAcβ1-3Galβ1-4GlcNAc | +++ | +++ | + | +++ | +++ | +++ | +++ | +++ |
| 23 | Neu5Acα2-3Galβ1-3GlcNAcβ1-3Galβ1-3GlcNAc |  | + |  | +++ | +++ | +++ | +++ | +++ |
| 24 | Neu5Acα2-3Galβ1-4GlcNAcβ1-3Galβ1-4GlcNAcβ1-3Galβ1-4GlcNAc | +++ | + | + | + | + | +++ | +++ | +++ |
| 25 | Neu5Acα2-3Galβ1-4GlcNAcβ1-3Galβ1-3GlcNAc |  | + |  | +++ | +++ | +++ | +++ | +++ |
| 26 | Neu5Acα2-3Galβ1-3GalNAc |  |  |  | +++ | +++ | +++ | + | +++ |
| 27 | Galβ1-3(Neu5Acα2-3Galβ1-4(Fucα1-3)GlcNAcβ1-6)GalNAc |  |  |  |  |  | + |  | +++ |
| 28 | Neu5Acα2-3Galβ1-3(Fucα1-4)GlcNAc |  | + | +++ | +++ | +++ | +++ | + | +++ |
| 29 | Neu5Acα2-3Galβ1-4(Fucα1-3)GlcNAc |  | +++ | +++ | +++ | +++ | +++ | +++ | +++ |
| 30 | Neu5Acα2-3Galβ1-4(Fucα1-3)GlcNAc |  | +++ | +++ |  |  | +++ | +++ | +++ |
| 31 | Neu5Acα2-3Galβ1-4(Fucα1-3)GlcNAcβ1-3Gal |  | +++ | +++ | +++ | +++ | +++ | +++ | +++ |
| 32 | Neu5Acα2-3Galβ1-4(Fucα1-3)GlcNAcβ1-3Galβ1-4GlcNAc |  | +++ | +++ | +++ | +++ | +++ | +++ | +++ |
| 33 | Neu5Acα2-3Galβ1-4(Fucα1-3)GlcNAcβ1-3Galβ1-4(Fucα1-3)GlcNAcβ1­3Galβ1-4(Fucα1-3)GlcNAc |  | +++ | +++ | +++ | +++ | +++ | +++ | +++ |
| 34 | Neu5Acα2-3Galβ1-4GlcNAcβ1-3Galβ1-4(Fucα1-3)GlcNAc |  |  |  |  |  |  |  |  |
| 35 | Neu5Acα2-3(GalNAcβ1-4)Galβ1-4GlcNAc |  |  |  |  |  |  |  |  |
| 36 | Neu5Acα2-3(GalNAcβ1-4)Galβ1-4GlcNAc |  |  |  |  |  |  |  |  |
| 37 | Neu5Acα2-3(GalNAcβ1-4)Galβ1-4Glc |  |  |  |  |  |  |  |  |
| 38 | Galβ1-3GalNAcβ1-4(Neu5Acα2-3)Galβ1-4Glc |  |  |  |  |  |  |  |  |
| 39 | Fucα1-2Galβ1-3GalNAcβ1-4(Neu5Acα2-3)Galβ1-4Glc |  |  |  |  |  |  |  |  |
| 40 | Fucα1-2Galβ1-3GalNAcβ1-4(Neu5Acα2-3)Galβ1-4Glc |  |  |  |  |  |  |  |  |
| 41 | Neu5Acα2-6Galβ1-4[6OSO3]GlcNAc |  |  |  |  |  |  |  |  |
| 42 | Galβ1-4GlcNAcβ1-2Manα1-3(Neu5Acα2-6Galβ1-4GlcNAcβ1-2Manα1­6)Manβ1-4GlcNAcβ1-4GlcNAc |  |  |  |  |  |  |  |  |
| 43 | GlcNAcβ1-2Manα1-3(Neu5Acα2-6Galβ1-4GlcNAcβ1-2Manα1-6)Manβ1­4GlcNAcβ1-4GlcNAc |  |  |  |  |  |  |  |  |
| 44 | Galβ1-4GlcNAcβ1-2Manα1-3(Neu5Acα2-6Galβ1-4GlcNAcβ1-2Manα1­6)Manβ1-4GlcNAcβ1-4GlcNAc |  |  |  |  |  |  |  |  |
| 45 | Neu5Acα2-6Galβ1-4GlcNAcβ1-2Manα1-3(GlcNAcβ1-2Manα1-6)Manβ1­4GlcNAcβ1-4GlcNAc |  |  |  |  |  |  |  |  |
| 46 | Neu5Acα2-6Galβ1-4GlcNAcβ1-2Manα1-3(Neu5Acα2-6Galβ1-4GlcNAcβ1­2Manα1-6)Manβ1-4GlcNAcβ1-4GlcNAc |  | +++ |  |  |  |  |  |  |
| 47 | Neu5Acα2-6Galβ1-4GlcNAcβ1-2Manα1-3(Neu5Acα2-6Galβ1-4GlcNAcβ1­2Manα1-6)Manβ1-4GlcNAcβ1-4GlcNAc |  | +++ |  |  |  | + |  | +++ |
| 48 | Neu5Acα2-6Galβ1-4GlcNAcβ1-2Manα1-3(Neu5Acα2-6Galβ1-4GlcNAcβ1­2Manα1-6)Manβ1-4GlcNAcβ1-4GlcNAc |  | +++ |  |  |  |  |  |  |
| 49 | Neu5Acα2-6Galβ1-4GlcNAcβ1-2Manα1-3(Galβ1-4GlcNAcβ1-2Manα1­6)Manβ1-4GlcNAcβ1-4GlcNAc |  |  |  |  |  |  |  |  |
| 50 | Neu5Acα2-6GalNAc |  |  |  |  |  |  |  |  |
| 51 | Neu5Acα2-6Gal |  |  |  |  |  |  |  |  |
| 52 | Neu5Acα2-6Galβ1-4Glc |  | + |  |  |  |  |  |  |
| 53 | Neu5Acα2-6Galβ1-4GlcNAc |  |  |  |  |  |  |  |  |
| 54 | Neu5Acα2-6Galβ1-4GlcNAc |  |  |  |  |  |  |  |  |
| 55 | Neu5Acα2-6GalNAcβ1-4GlcNAc |  |  |  |  |  |  |  |  |
| 56 | Neu5Acα2-6Galβ1-4GlcNAcβ1-3Galβ1-4GlcNAc |  | + |  |  |  |  |  | +++ |
| 57 | Neu5Acα2-6Galβ1-4GlcNAcβ1-3Galβ1-4(Fucα1-3)GlcNAcβ1-3Galβ1­4(Fucα1-3)GlcNAc |  |  |  |  |  |  |  |  |
| 58 | Galβ1-3(Neu5Acα2-6)GlcNAcβ1-3Galβ1-4Glc | +++ | +++ |  |  |  |  |  |  |
| 59 | Galβ1-3(Neu5Acα2-6)GalNAc |  |  |  |  |  |  |  |  |
| 60 | Neu5Acα2-3Galβ1-4GlcNAcβ1-2Manα1-3(Neu5Acα2-6Galβ1-4GlcNAcβ1­2Manα1-6)Manβ1-4GlcNAcβ1-4GlcNAc | +++ | +++ |  | +++ | +++ | +++ | +++ | +++ |
| 61 | Neu5Acα2-6Galβ1-4GlcNAcβ1-2Manα1-3(Neu5Acα2-3Galβ1-4GlcNAcβ1­2Manα1-6)Manβ1-4GlcNAcβ1-4GlcNAc | +++ | +++ |  | +++ | +++ | +++ | +++ | +++ |
| 62 | Neu5Acα2-3Galβ1-3(Neu5Acα2-6)GalNAc |  | +++ |  | +++ | +++ | +++ | +++ | +++ |
| 63 | Neu5Acα2-3Galβ1-3(Neu5Acα2-6)GalNAc | +++ | +++ | + |  |  | +++ | +++ | +++ |
| 64 | Neu5Acα2-3(Neu5Acα2-6)GalNAc | + | +++ |  |  |  |  |  | +++ |
| 65 | Neu5Gc |  |  |  |  |  |  |  |  |
| 66 | Neu5Gcα2-3Galβ1-3(Fucα1-4)GlcNAc |  |  |  | + | + | +++ |  | +++ |
| 67 | Neu5Gca2-3Galβ1-3GlcNAc |  |  |  | +++ | +++ | +++ |  | +++ |
| 68 | Neu5Gcα2-3Galβ1-4(Fucα1-3)GlcNAc |  |  | + | +++ | +++ | +++ |  | +++ |
| 69 | Neu5Gcα2-3Galβ1-4GlcNAc |  |  | + | +++ | +++ | +++ |  | +++ |
| 70 | Neu5Gcα2-3Galβ1-4Glc |  |  |  | +++ | +++ | +++ |  | +++ |
| 71 | Neu5Gcα2-6GalNAc |  |  |  |  |  |  |  |  |
| 72 | Neu5Gcα2-6Galβ1-4GlcNAc |  |  |  |  |  |  |  |  |
| 73 | Neu5Acα2-8Neu5Ac |  |  |  |  |  |  |  |  |
| 74 | Neu5Acα2-8Neu5Acα2-8Neu5Ac |  |  |  |  |  |  |  |  |
| 75 | Neu5Acα2-8Neu5Acα2-3(GalNAcβ1-4)Galβ1-4Glc |  |  |  |  |  |  |  |  |
| 76 | Neu5Acα2-8Neu5Acα2-3Galβ1-4Glc |  |  |  |  |  |  |  |  |
| 77 | Neu5Acα2-8Neu5Acα2-8Neu5Acα2-3(GalNAcβ1-4)Galβ1-4Glc |  |  |  |  |  |  |  |  |
| 78 | Neu5Acα2-8Neu5Acα2-8Neu5Acα2-3Galβ1-4Glc |  |  |  |  |  |  |  | + |
| 79 | Neu5Acα2-8Neu5Ac |  |  |  |  |  |  |  |  |
| 80 | Neu5Acα2-8Neu5Ac |  |  |  |  |  |  |  |  |
| 81 | Neu5Acα2-8Neu5Acα2-8Neu5Ac |  |  |  |  |  |  |  |  |
| 82 | Neu5Acβ2-6GalNAc |  |  |  |  |  |  |  |  |
| 83 | Neu5Acβ2-6Galβ1-4GlcNAc |  |  |  |  |  |  |  |  |
| 84 | Neu5Gcβ2-6Galβ1-4GlcNAc |  |  |  |  |  |  |  |  |
| 85 | Galβ1-3(Neu5Acβ2-6)GalNAc |  |  |  |  |  |  |  |  |
| 86 | 9NAcNeu5Ac |  |  |  |  |  |  |  |  |
| 87 | 9NAcNeu5Acα2-6Galβ1-4GlcNAc |  |  |  |  |  |  |  |  |
| 88 | Galβ1-4GlcNAcβ1-3Galβ1-4GlcNAcβ1-3Galβ1-4GlcNAc |  |  |  |  |  |  |  |  |
| 89 | Galβ1-3GlcNAcβ1-3Galβ1-3GlcNAc |  |  |  |  |  |  |  |  |
| 90 | Fucα1-2Galβ1-3GlcNAcβ1-3Galβ1-4Glc |  |  |  |  |  | + |  |  |
| 91 | Fucα1-2Galβ1-4(Fucα1-3)GlcNAcβ1-3Galβ1-4(Fucα1-3)GlcNAc |  |  |  |  |  |  |  |  |
| 92 | GalNAcα1-3(Fucα1-2)Galβ1-3GlcNAc |  |  |  |  |  |  |  |  |
| 93 | GalNAcα1-3(Fucα1-2)Galβ1-4GlcNAc |  |  |  |  |  |  |  |  |
| 94 | Galα1-3(Fucα1-2)Galβ1-3GlcNAc |  |  |  |  |  |  |  |  |
| 95 | Galα1-3(Fucα1-2)Galβ1-4(Fucα1-3)GlcNAc |  |  |  |  |  |  |  |  |
| 96 | Galβ1-3GalNAc |  |  |  |  |  |  |  |  |
